# Pre-pandemic cross-reactive antibody and cellular responses against SARS-CoV-2 among female sex workers in Dakar, Senegal

**DOI:** 10.3389/fpubh.2025.1522733

**Published:** 2025-01-23

**Authors:** Bobby Brooke Herrera, Beth Chaplin, Souleymane MBoup, Adam Abdullahi, Michelle He, Sydney M. Fisher, Sulaimon Akanmu, Charlotte A. Chang, Donald J. Hamel, Ravindra K. Gupta, Phyllis J. Kanki

**Affiliations:** ^1^Rutgers Global Health Institute, Rutgers University, New Brunswick, NJ, United States; ^2^Department of Medicine, Division of Allergy, Immunology, and Infectious Diseases, and Child Health Institute of New Jersey, Rutgers Robert Wood Johnson Medical School, Rutgers University, New Brunswick, NJ, United States; ^3^Mir Biosciences, Inc., Dunellen, NJ, United States; ^4^Department of Immunology and Infectious Diseases, Harvard T.H. Chan School of Public Health, Boston, MA, United States; ^5^Institut De Recherche En Santé De Surveillance Épidémiologique Et De Formation (IRESSEF), Dakar, Senegal; ^6^Cambridge Institute of Therapeutic Immunology and Infectious Disease (CITIID), Cambridge, United Kingdom; ^7^Department of Medicine, University of Cambridge, Cambridge, United Kingdom; ^8^Lagos University Teaching Hospital, College of Medicine, University of Lagos, Lagos, Nigeria

**Keywords:** SARS-CoV-2, antibody, cellular responses, cross-reactivity, Senegal

## Abstract

**Background:**

The COVID-19 pandemic had a severe impact globally, yet African populations exhibited unexpectedly lower rates of severe disease and mortality. We investigated the potential role of pre-existing immunity in shaping the epidemiology of COVID-19 in Africa.

**Methods:**

Plasma collected from Senegalese female sex workers prior to the COVID-19 pandemic was screened for SARS-CoV-2 and human coronavirus (hCoV) antibodies by virion immunoblots. For antibody-reactive plasma, paired peripheral blood mononuclear cells were stimulated by fusion proteins and IFN-*γ* cellular responses were assessed via ELISPOT.

**Results:**

We observed substantial levels of pre-existing cross-reactive immunity to SARS-CoV-2, stemming from prior exposure to seasonal hCoVs. Our antibody analysis revealed a 23.5% (47/200) seroprevalence rate against SARS-CoV-2 nucleocapsid (N). These samples were then probed for antibodies against hCoV spike (S) and/or N antigens; 85.1% (40/47), 70.2% (33/47), and 95.7% (45/47) were antibody reactive against hCoV-229E, hCoV-OC43, or hCoV-HKU1, respectively. Our analysis of cellular responses also demonstrated cross-reactivity to SARS-CoV-2 with 80.0% (36/45) and 82.2% (37/45) showing IFN-*γ* responses against S and N, respectively. A unique pre-pandemic subject had cross-reactive SARS-CoV-2 S antibodies with detectable neutralization and cross-reactive cellular responses.

**Conclusion:**

These findings suggest that prior hCoV exposure may induce cross-reactive adaptive immunity, potentially contributing to protection against COVID-19. Our study provides unique data on the dynamics of hCoV and SARS-CoV-2 immunity in Senegal and underscores the importance of understanding the role of pre-existing immunity in shaping COVID-19 outcomes globally.

## Introduction

The first COVID-19 cases were recorded on the African continent in March 2020, yet by the end of the pandemic in May 2023, Africa’s reported SARS-CoV-2 infections and deaths constituted only 2–4% of the global disease burden despite being home to ~17% of the world’s population. Early projections of the COVID-19 pandemic’s impact on Africa predicted massive loss of life. Most African countries lacked adequate healthcare infrastructure, workforce, and equipped facilities to cope with a novel and highly infectious respiratory pathogen. However, other characteristics of most African countries suggested different outcomes from what was seen in resource-rich settings (1). While COVID-19 disease severity was strongly correlated with older age, Africa has a significantly younger population age distribution with lower rates of co-existing morbidities compared with other continents. Africa’s environmental factors and contact structures, such as time spent outside, may also limit disease spread. Additionally, pre-existing immunity from exposure to related viruses may explain the unexpected low rates of severe disease and mortality observed across the continent (2).

In 2019, SARS-CoV-2 emerged as a new human coronavirus, even though worldwide populations were known to be regularly exposed to seasonal human coronaviruses (hCoVs) responsible for the “common cold,” including *α*-coronaviruses (hCoV-229E, hCoV-NL63,) and *β*-coronaviruses (hCoV-OC43, hCoV-HKU1). Although associated with mild illness, these hCoVs are classified in the same *Coronaviridae* family as the SARS coronaviruses, which include SARS-CoV-1, SARS-CoV-2, and Middle East Respiratory Syndrome (MERS) and share genetic and structural similarities. hCoVs are globally endemic and estimated to be responsible for up to 15–30% of pre-pandemic annual respiratory infections, which are seasonal (e.g., fall and winter) and most prevalent, though undercounted, in young children (3).

The widespread circulation of hCoVs with repeated exposure of human populations and shared sequence homology to SARS-CoV-2 led to the suggestion that hCoV immunologic memory could result in cross-reactive cellular and humoral responses that might in part explain the heterogeneity of COVID-19 presenting symptoms and pathogenic outcomes. While not providing sterilizing immunity, pre-existing hCoV immunity could reduce transmission and ameliorate symptoms of the related SARS-CoV-2. Therefore, research has been conducted to determine the role of pre-existing cross-reactive immunity to SARS-CoV-2.

Multiple US and European studies described CD4^+^ T cell reactivity in SARS-CoV-2 unexposed individuals, attributed to pre-existing memory responses to hCoVs (3–7). In a UK household study, exposed contacts that remained PCR-negative showed significantly higher frequency of cross-reactive (*p* = 0.0139) and nucleocapsid (N)-specific IL-2 secreting memory T cells (*p* = 0.0355) (4). Swadling et al. (5) in a study of potentially abortive SARS-CoV-2 infection in UK healthcare workers (HCWs) described pre-existing T cell reactivity directed against the early transcribed replication-transcription complex (RTC). The RTC, consisting of RNA polymerase co-factor non-structural protein 7 (NSP7), RNA polymerase NSP12 and helicase NSP13, is expressed early in the viral life cycle and is highly conserved among members of *Coronaviridae* (5). Studies of pre-pandemic samples collected from Africa have also demonstrated cross-reactive T cell responses against several SARS-CoV-2 antigens (6, 7). These studies have led to the hypothesis that prior hCoV infection might provide protective cross-reactive memory, perhaps more likely in younger individuals in whom infections with hCoVs are more prevalent and recent. As described by Lipsitch et al. (8) pre-existing cross-reactive memory T cells could lower respiratory tract viral load thereby limiting the duration and severity of disease with potential to reduce viral spread.

Pre-existing antibody studies have also described distinct humoral responses to SARS-CoV-2 associated with prior hCoV infection. Ng et al. described uninfected donors with IgG reactivity to conserved epitopes in the S2 subunit of the SARS-CoV-2 spike (S) protein distinct from *de novo* humoral responses in infected individuals that targeted both S1 and S2 subunits with concomitant IgM and IgA responses (9). Multiple studies conducted in the US and Europe have identified antibodies to hCoVs (OC43) that were associated with lower risk of severe COVID-19 (10, 11).

Over the course of the COVID-19 pandemic in Africa, studies based on SARS-CoV-2 antibody detection described high prevalence rates of infection, in contrast with surveillance data (12). Yet, even with anticipated under testing and underreporting, severe COVID-19 cases and mortality remained unexpectedly low, especially in the general population (13, 14). While multiple factors are likely to contribute to the capacity of individuals to generate cross-reactivity, it is possible that the order and composition of different infections may play a role in determining the efficacy of the immune response in preventing symptomatic or severe COVID-19. Currently, high rates of SARS-CoV-2 infection and COVID-19 vaccine exposure globally challenge the ability to investigate cross-reactive responses in individuals who have not been exposed to SARS-CoV-2.

While our research group has reported on SARS-CoV-2 immune responses in Nigeria (6), in this study we leveraged paired plasma and peripheral blood monocular cell (PBMC) samples collected in Senegal, West Africa prior to the COVID-19 pandemic. Our objectives were to measure hCoV seroprevalence rates and to characterize the pre-existing cross-reactive antibody and cellular responses to SARS-CoV-2 in our pre-pandemic West African samples. The hypothesis was that prior hCoV infection may induce cross-reactive immune responses to SARS-CoV-2. Detection of cross-reactive immunity would suggest further studies to investigate whether and how pre-existing hCoV immunity might confer protection against severe COVID-19 disease in Africa.

## Materials and methods

### Human samples

This study tested 200 archived plasma samples and paired PBMCs (45 matched to antibody-reactive plasma and 20 to negative plasma to serve as controls) collected from 2004 to 2005 in our multi-decade prospective cohort study of HIV-1 and HIV-2 in Senegalese female sex workers (FSWs) (15–17). Self-identified FSW routinely visiting a health care clinic in Dakar provided blood samples to test for multiple sexually transmitted infections, including HIV-1 and HIV-2, and were examined clinically; excess blood samples were de-identified and archived with their corresponding clinical data. More than 80% of women sampled were HIV negative and most had paired plasma and liquid nitrogen preserved PBMCs with accompanying clinical and immunologic data at more than 2 timepoints. The viability and functional integrity of these cryopreserved PBMCs have been previously demonstrated in our studies of HIV and flavivirus T-cell immunity (18, 19). At the time of the original study, all women provided informed consent for the collection and archiving of samples and corresponding data; ethical clearance from the Harvard Institutional Review Board (IRB) and the research ethics committee at Chiekh Anta Diop University, Dakar, Senegal were obtained. All archived samples and corresponding data were anonymized, and as such this secondary analysis did not constitute human subject research by institutional guidelines and regulations which follow the Federal Policy for Protection of Human Subjects.

### Virion-based and RecomLine immunoblots

We screened plasma samples by immunoblot assay on virion preparations from SARS-CoV-2 or hCoVs. Briefly, Vero E6 cells or susceptible cell line were infected with SARS-CoV-2 (Isolate USA-WA1/2020, BEI Resources NR-52281) or hCoVs (OC43, NL63, 229E, BEI Resources NR-5621, 470 and 52,726, respectively) and propagated for 5 days. Supernatants were clarified at 10,000 g for 20 min at 4°C, precipitated with PEG-8000 and NaCl, and then resolved by sucrose gradient ultracentrifugation at 170,000 g for 90 min at 4°C. Viral pellets were lysed with complete NP40 buffer containing protease inhibitors. Viral lysates were added to non-reducing buffer (final concentrations of 2% SDS, 0.5 M Tris pH 6.8, 20% glycerol, 0.001% bromophenol blue) and subjected to 12% PAGE and Western blot analysis using patient serum (1:250) as primary antibody and anti-human IgG horseradish peroxidase (HRP) (1:2,000; ThermoFisher Scientific, Waltham, MA) as secondary antibody. Visualization was performed using Metal Enhanced DAB Substrate Kit (ThermoFisher Scientific, Waltham, MA) per the manufacturer’s instructions.

The RecomLine SARS-CoV-2 IgG (Mikrogen) is a CE-marked immunoblot assay which determines the IgG responses towards the recombinant N antigens of the seasonal hCoV 229E, OC43, NL63 and HKU-1 in parallel to SARS-CoV-2 N, receptor-binding domain (RBD), and S1 antigens (20). This assay was used to confirm antibody responses in the single individual that initially screened antibody reactive to SARS-CoV-2 S-only.

All samples were probed for antibodies against SARS-CoV-2, samples that were positive for SARS-CoV-2 N-only were then further probed for antibodies against hCoV-229E and hCoV-OC43 S and N proteins. Antibodies to hCoV-HKU1 S and N proteins were determined by immunoblot of recombinant proteins as described (S protein, NR-5373) or RecomLine (N protein, Mikrogen).

### ELISPOT assay

ELISPOT assays were conducted as described previously (18, 21, 22). In brief, 200,000 PBMCs per well were seeded in duplicate into 96-well plates coated with the anti-human IFN-*γ* BD Capture Antibody (Catalog number: 551873, Becton Dickinson (BD), Franklin Lakes, NJ). The cells were stimulated with fusion proteins consisting of a modified version of the *Bacillus anthracis* lethal factor (LFn) and SARS-CoV-2 S or N (Mir Biosciences, Inc., Dunellen, NJ) and either a negative (LFn alone, Mir Biosciences, Inc., Dunellen, NJ) or positive (PHA, ThermoFisher, Waltham, MA) control at 37°C with 5% CO_2_ for 24 h. LFn has been shown to transport protein cargoes into the cell cytosol for MHC Class I and II processing, inducing T cell responses (6, 18, 21, 22). After incubation, plates were washed followed by incubation for 2 h at room temperature with the BD Detection Antibody (Catalog number: 551873, BD, Franklin Lakes, NJ). Subsequently, plates were washed and incubated with streptavidin (BD, Franklin Lakes, NJ) for 1 h at room temperature. Finally, plates were washed and incubated with 3-amino-9-ethyl-carbazole substrate (Catalog number: 551951, BD, Franklin Lakes, NJ) for 30 min. IFN-*γ* spot forming cells (SFC) were counted using the ImmunoSpot S6 Ultra M2 Analyzer (ImmunoSpot, Shaker Heights, OH) (18, 21, 22).

### Virus neutralization titer analyses

Pseudotype virus neutralization assay was performed on Hela-ACE2 cells using SARS-CoV-2 spike pseudotype virus (PV) expressing luciferase as previously described (23, 24). Briefly, dried plasma spots were eluted and heat inactivated at 54°C for 1 h and incubated with PVs at 37°C for 1 h prior to addition of Hela-ACE2 cells. The plasma dilution/virus mix was incubated for 48 h in a 5% CO_2_ environment at 37°C, and luminescence was measured using the Bright-Glo Luciferase assay system (Promega UK, United Kingdom). Neutralization was calculated relative to the virus and cell only controls. Data was analyzed in GraphPad Prism v9.3.1 where 50% neutralization (ID_50_) values were calculated and the limit of detection for neutralization was set at ID_50_ of 20 units.

### Statistical analyses

SARS-CoV-2 and hCoV seroprevalences were quantified as proportions of positive over total tested. Bivariate analyses explored associations between select demographic characteristics of the women with available data and SARS-CoV-2 N-reactive immunoblot results. Demographic characteristics included: year of sampling, age at sampling, neighborhood, ethnicity, country of origin, marital status, religion, education, and total children. The chi-square test was used to obtain *p*-values to detect statistically significant associations, and Fisher’s exact test for categories with observed frequencies ≤five. Stata 15 (StataCorp LLC, College Station, TX, United States) was used for the analyses.

For cellular responses, a positive response was defined as any test well with an IFN-*γ* SFC count ≥3 times and at least 3 standard deviations greater than the mean IFN-*γ* SFC count of the negative control wells. We calculated the proportion of PBMC samples showing cross-reactivity to SARS-CoV-2 S and N proteins among the SARS-CoV-2 N-only group with any hCoV S + N/S antibodies and the negative control group. The mean IFN-γ SFC counts between the two groups were compared using the non-parametric Mann–Whitney U test. For all analyses, statistical significance was defined at a level of *p* < 0.05.

## Results

### Pre-pandemic cross-reactive SARS-CoV-2 antibody responses and hCoV seroprevalence rates

**Figure 1:**
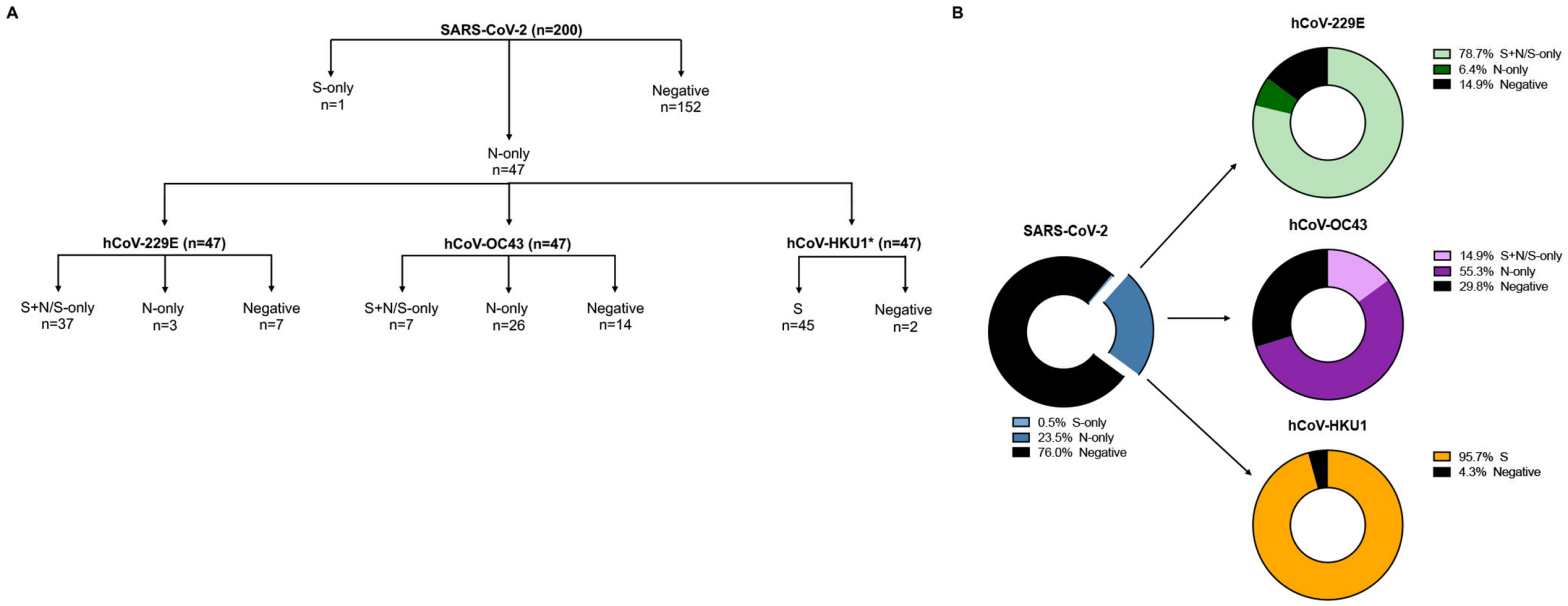
SARS-CoV-2 and hCoVs seroprevalence rates. **(A)** Schematic representation of serology workflow. *, Samples were analyzed against hCoV-HKU1 S only. **(B)** 200 samples were initially tested against SARS-CoV-2 S and N; 47 samples had antibodies against SARS-CoV-2 N-only (blue donut chart). These 47 samples were then tested against hCoV-229E S and N (green donut chart), hCoV-OC43 S and N (purple donut chart), and hCoV-HKU1 S (yellow donut chart); only a subset of samples were tested against hCoV-HKU1 N.

**Figure 2:**
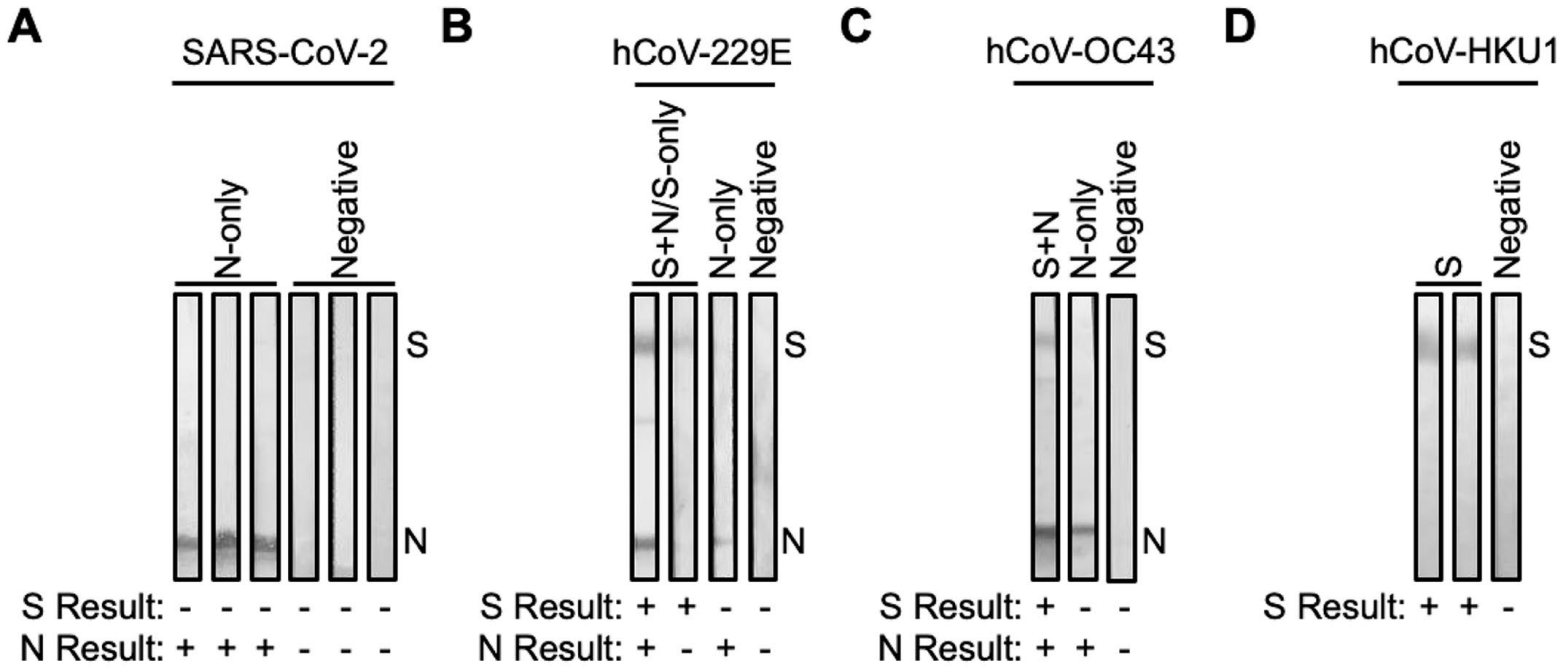
SARS-CoV-2 and hCoV antibody cross-reactive in samples collected pre-pandemic. Plasma collected from FSWs prior to the COVID-19 pandemic were subjected to immunoblot analysis. Representative images of immunoblots for samples that had antibodies for **(A)** SARS-CoV-2 N-only or were negative, **(B)** hCoV-229E S + N/S-only, N-only, or were negative, **(C)** hCoV-OC43 S + N, N-only, or were negative, and **(D)** hCoV-HKU1 S or were negative.

Pre-pandemic plasma samples (*n* = 200) from the Senegalese FSW cohort were analyzed for the presence of antibodies directed against SARS-CoV-2 S and/or N. 152 of 200 (76%) were negative, while 47 (23.5%) showed reactivity to SARS-CoV-2 N-only, suggestive of prior exposure to hCoVs (Figures 1, 2A). To determine hCoV prevalence rates, the 47 samples reactive to SARS-CoV-2 N-only were analyzed for antibodies directed against hCoV-229E S and N, hCoV-OC43 S and N, and hCoV-HKU1 S. Of the samples tested, 37 (78.7%) samples showed reactivity to hCoV-229E S ± N, seven (14.9%) to hCoV-OC43 S ± N, and 45 (95.7%) to hCoV-HKU1 S (Figures 1, 2B–D). Of the 37 hCoV-229E S ± N samples, 30 (81.1%) were reactive to S + N, while seven (18.9%) were reactive to S-only. Of the seven hCoV-OC43 S ± N samples, six (85.7%) were reactive to S + N, while one (14.3%) was reactive to S-only. Of note, 36 out of the 47 samples (76.6%) were reactive to both hCoV-229E S and hCoV-HKU1 S (Supplementary Table S2). Only six (12.8%) were reactive to S of hCoV-229E, hCoV-OC43, and hCoV-HKU1 (Supplementary Tables S1–S3).

### Risk factor analysis

The age range at sampling was 22 to 57 years, with median age 38 years (IQR: 32–44 years). Bivariate analysis of risk factors associated with SARS-CoV-2 N antibody reactivity included: year of sampling, older age at sampling, neighborhood, ethnicity, country of origin, marital status, religion, education, and total children. There was a potential association between SARS-CoV-2 N-reactivity and age > 44 years (the highest age quartile, *p* = 0·08) and with having ≥three children (*p* = 0·04) (Table 1). Women >44 years of age were more likely to have ≥three children.

**Table 1:** Associations between baseline characteristics and SARS-CoV-2 nucleocapsid antibody reactivity.

	N non-reactive, n (%)	N reactive, n (%)	Chi-square *p* value*
Year of sampling			
2004	74 (81.3%)	17 (18.7%)	0.14
2005	79 (72.5%)	30 (27.5%)	
Age at sampling			
≤ 44 years	92 (76.0%)	29 (24.0%)	0.08
> 44 years	22 (61.1%)	14 (38.9%)	
Neighborhood			
Dakar	82 (75.2%)	27 (24.8%)	0.58
Outside Dakar	37 (71.2%)	15 (28.8%)	
Ethnicity			
Wolof	47 (78.3%)	13 (21.7%)	0.19
Other	68 (68.7%)	31 (31.3%)	
Country of origin			
Senegal	106 (73.1%)	39 (26.9%)	0.54*
Other	9 (64.3%)	5 (35.7%)	
Marital status			
Single	34 (77.3%)	10 (22.7%)	0.58*
Divorced	64 (71.9%)	25 (28.1%)	
Widowed	7 (63.6%)	4 (36.4%)	
Religious identification			
Muslim	94 (73.4%)	34 (26.6%)	0.77*
Christian	11 (68.8%)	5 (31.3%)	
Education			
None	43 (69.4%)	19 (30.6%)	
1–6 years	30 (81.1%)	7 (18.9%)	0.43
7–14 years	30 (71.4%)	12 (28.6%)	
Total children			
0–2	70 (78.7%)	19 (21.3%)	0.04
≥ 3	45 (64.3%)	25 (35.7%)	

Of 200 samples tested, 47 were SARS-CoV-2 N positive and 153 were SARS-CoV-2 N negative. 159 had a corresponding baseline clinical record. N = nucleocapsid. ^*^Fisher’s exact test was used to obtain p values for variables with cells with observed frequencies ≤ 5.

### Pre-pandemic cross-reactive T-cell responses to SARS-CoV-2

**Figure 3:**
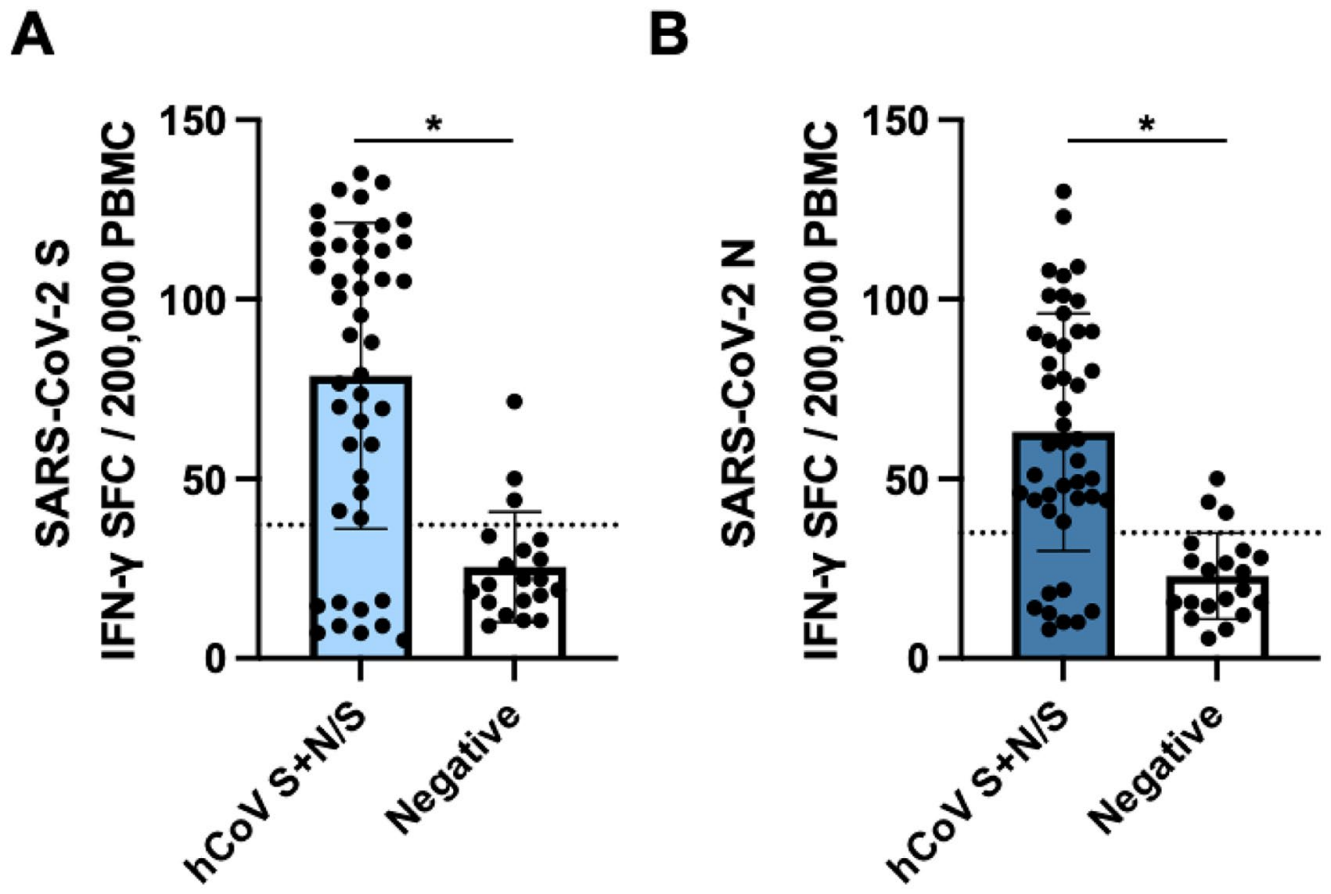
Cross-reactive SARS-CoV-2 cellular responses in samples collected pre-pandemic. PBMCs collected from FSWs prior to the COVID-19 pandemic were stimulated with **(A)** LFn-SARS-CoV-2 S or **(B)** LFn-SARS-CoV-2 N and IFN-*γ* responses were detected by ELISPOT. SFC, spot forming cells. Black dotted lines, LFn-SARS-CoV-2 S and N cutoffs. Cells were stimulated with LFn alone as a negative control and with PHA as a positive control.

Of the 47 pre-pandemic samples that showed antibody reactivity to SARS-CoV-2 N-only and also antibody reactivity to hCoV S + N/S, 45 paired PBMCs were available, along with 20 paired PBMCs from negative controls, and were analyzed for cross-reactive IFN-*γ* cellular responses against SARS-CoV-2 S and N. Among SARS-CoV-2 N-reactives, 36 (80.0%) showed IFN-*γ* reactivity against SARS-CoV-2 S (Figure 3A) while nine (20.0%) were negative. Similarly, 37 (82.2%) showed IFN-γ reactivity against SARS-CoV-2 N (Figure 3B) while eight (17.8%) were negative. Among the 20 antibody negative controls, three (15.0%) demonstrated weak IFN-γ cellular reactivity against both SARS-CoV-2 S and N. SARS-CoV-2 S and N cellular responses measured by mean IFN-γ SFCs were correlated with hCoV antibody reactivity (*p* < 0.0001).

### Evidence for potential pre-pandemic SARS-CoV-2 variant

**Figure 4:**
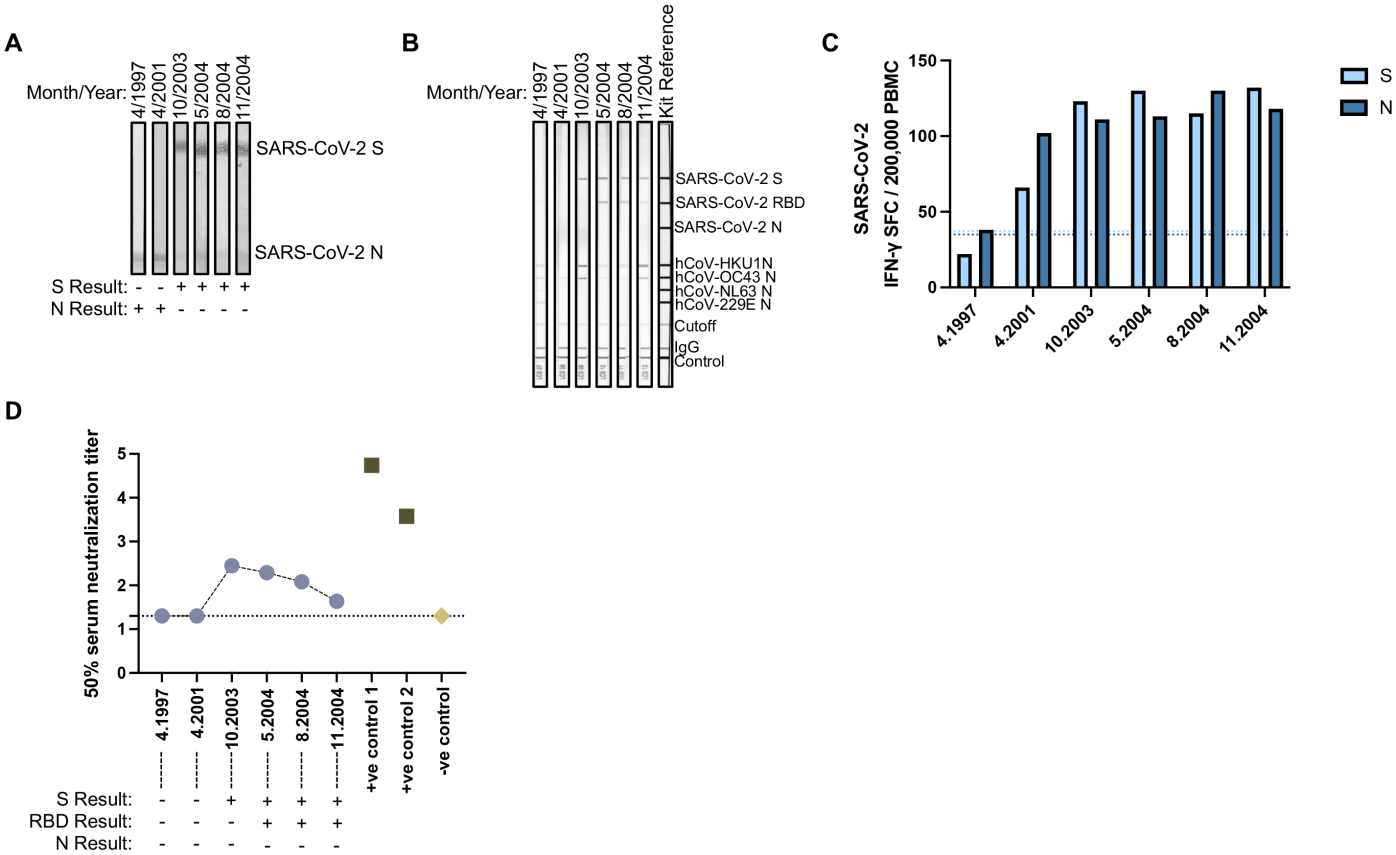
Evidence for pre-pandemic SARS-CoV-2 variant. Longitudinally collected plasma samples collected from a FSW between 1997 and 2004 were subjected to **(A)** SARS-CoV-2 immunoblots or the commercial **(B)** Mikrogen assay. **(C)** Paired PBMCs samples were stimulated with LFn-SARS-CoV-2 S or LFn-SARS-CoV-2 N and IFN-γ responses were detected by ELISPOT. SFC, spot forming cells. Light blue dotted line, LFn-SARS-CoV-2 S cutoff. Dark blue dotted line, LFn-SARS-CoV-2 N cutoff. **(D)** Plasma neutralization (Log10) of pseudotype virus against SARS COV-2 Wild type (Wu-1) in one individual with longitudinal samples collected between 1997 and 2004 including two positive controls from SARS CoV-2 infected and vaccinated adults in 2021 and pre-pandemic human sera as negative control. Results displayed under the x-axis correspond to their antibody reactivity via immunoblot assays.

In our initial serology, a single sample collected from a FSW in November 2004 demonstrated antibody reactivity to SARS-CoV-2 S-only (Figure 1). Longitudinal antibody analysis of six samples collected from this individual between 1997 to 2004 was conducted by SARS-CoV-2 immunoblot as well as the Mikrogen assay that detected antibodies against the individual antigens of SARS-CoV-2 (S, RBD, and N) and hCoVs (HKU1, OC43, NL63, and 229E N). Samples collected in 1997 and 2001 showed antibody reactivity to SARS-CoV-2 N-only by immunoblot with weak antibodies against hCoVs, particularly in the 1997 sample (Figures 4A,B). Seroconversion to SARS-CoV-2 S (2003) and S and RBD (May to November 2004) was observed by both immunoblot and the Mikrogen assay, accompanied with stronger reactivity to hCoV-HKU1 N and hCoV-OC43 N. This seroprofile is unique from our other pre-pandemic serology and may indicate exposure to a novel variant—one more closely related to SARS-CoV-2 – or potentially cross-reactivity to other pathogens. Additionally, longitudinal cellular responses were analyzed between 1997 and 2004. In 1997, this individual only had cellular responses against SARS-CoV-2 N. By 2001, this individual had cellular responses against both SARS-CoV-2 S and N that persisted throughout 2004 (Figure 4C). Sequential analysis of SARS-CoV-2 neutralization revealed no detectable neutralization response against SARS-COV-2 wild-type (Wu-1614G) in samples collected in 1997 and 2001 but showed detectable neutralization response in samples collected in October 2003 (ID_50_ titer = 281.3) with waning responses observed in May 2004 (ID_50_ titer = 195.6); August 2004 (ID_50_ titer = 121.1) and November 2004 (ID_50_ titer = 43.3). The observation of detectable responses coincided with seroconversion of S and RBD antibodies reflecting exposure to hCoVs with possible cross-reactive potential due to sequence homogeneity to a SARS-CoV-2 variant. Clinical examination data collected as part of the prospective HIV clinical protocol did not indicate reporting of symptoms or signs of respiratory disease, but the patient was noted to have lost significant (>10%) weight between 2001 to 2003, during which S antibody seroconversion occurred. The patient was also HIV-positive, 43 years of age in 2003, and had three living children.

## Discussion

In early 2021, we studied the immune responses in Nigerian healthcare workers (HCWs) at Lagos University Teaching Hospital (*n* = 134) and in Oxford/AstraZeneca COVID-19 vaccine recipients from the general population (*n* = 116) across five local government areas (LGAs) in Lagos state (6). Antibodies directed to only SARS-CoV-2 N in the absence of S antibody reactivity were detected in 9.7% (13/134) of HCWs and 15.5% (18/116) of the general population. This antibody profile directed to the SARS-CoV-2 N alone was suggestive of pre-existing cross-reactive immunity stemming from prior hCoV infection. To investigate this possibility further, in this study we tested archived plasma samples collected before the COVID-19 pandemic from our FSW cohort in Dakar, Senegal (2004–2005), and observed 23.5% (47/200) of individuals with pre-existing immunity to SARS-CoV-2 N-only. Further testing demonstrated prior infections with the seasonal hCoVs, providing evidence that the SARS-CoV-2 N-only reactivity potentially resulted from cross-reactivity from prior hCoV exposure. Genetic analysis has shown that the coronavirus N protein is more conserved than the S protein. As such, previously hCoV exposed individuals can demonstrate cross-reactive N antibodies without cross-reactive S antibodies (6, 25).

In one of the few studies conducted on samples from Africa prior to the COVID-19 pandemic, Tso et al. reported higher reactivity to hCoV N and S antigens with significantly higher rates of cross-reactivity in Zambia (14.1%) and Tanzania (19%) compared to the US (2.4%) (26). In a multi-national study, Pedersen et al. reported higher SARS-CoV-2 N reactivity in Gabon (17.2%) compared to reactivity in Canada (2.4%), Denmark (2.5%), and Brazil (3.7%). In a study conducted in Senegal, which predominantly included individuals under 17 years of age, high antibody cross-reactivity to both S and N antigens of SARS-CoV-2 was observed; however, these samples failed to demonstrate *in vitro* neutralization (25). A possible explanation for non-neutralizing antibodies in this study could be due to these individuals having been infected with a coronavirus that is less related to SARS-CoV-2. Regardless, while most of these earlier studies did not observe cross-neutralization against SARS-CoV-2, a more recent study of pre-pandemic Vietnamese samples showed neutralization against multiple SARS-CoV-2 strains (27). In our current study of pre-pandemic FSWs, we similarly detected antibody cross-reactivity to SARS-CoV-2, but we also concurrently detected cellular cross-reactivity to support the antibody results.

While most investigators study T cell responses to specific antigens using overlapping peptides, we have used full-length antigens or large peptides fused to a modified version of the *Bacillus anthracis* lethal factor (LFn). LFn has been shown to act as a molecular shuttle, transporting antigens of interest into the cytosol for MHC Class I and Class II processing for CD4^+^ and CD8^+^ T cell presentation. In the past, we have used LFn to study T cell responses against several viruses, including HIV, Ebola, Zika, dengue, and SARS-CoV-2 (6, 18, 21, 22). In our SARS-CoV-2 study of Nigerian HCWs and individuals from the general population, among individuals with SARS-CoV-2 N-only antibodies prior to vaccination, suggestive of prior exposure to hCoV, 81.8% (9/11) had T cell responses to SARS-CoV-2 N (6). These results provided further evidence that individuals who were previously exposed to hCoVs could have cross-reactive cellular responses against SARS-CoV-2.

Cross-reactive T cell responses play a protective role in protection against COVID-19. Cellular responses were shown to map to cross-reactive recognition of SARS-CoV-2 by T cells induced by hCoVs (28–30). SARS-CoV-2-specific T cells were also able to cross-react with SARS-CoV-1 and MERS-CoV (31, 32). Recently, Tarke et al. (33) demonstrated that pre-pandemic samples collected from healthy adults in the US with confirmed previous exposure to hCoV-NL63 and -OC43 had significant cross-reactive T cell responses to SARS-CoV-2, particularly to conserved regions of SARS-CoV-2 S, N, nsp3, and nsp12. Similarly, Namuniina et al. showed that in pre-pandemic samples collected in Uganda, a majority of subjects had both CD4^+^ and/or CD8^+^ T cell responses against SARS-CoV-2 S and non-S peptide pools (7). This study however did not determine whether pre-existing immunity based on previous exposure to hCoV was responsible for the cross-reactive cellular responses.

In the current study using pre-pandemic samples collected from Senegalese FSWs, we show evidence of cellular cross-reactivity to SARS-CoV-2 with 80.0% (36/45) and 82.2% (37/45) of PBMC samples matched to SARS-CoV-2 N antibody reactive plasma samples demonstrating IFN-*γ* responses against S and N, respectively. Although these N antibody reactive samples showed no S antibody reactivity, the majority demonstrated cross-reactive cellular responses to both SARS-CoV-2 S and N, suggesting that cellular responses against this less conserved region may be more sustained over time than humoral responses. Among the SARS-CoV-2 negative controls, a majority (17/20, 85.0%) did not have cellular responses. However, three control individuals showed weak cellular responses against SARS-CoV-2 S and N. This again may reflect the more temporal nature of humoral immunity compared with the longevity of cellular immunity. Additionally, the individual that initially tested antibody positive for SARS-CoV-2 S-only also had sustained cross-reactive cellular responses to SARS-CoV-2 S and/or N from 1997 to 2004 with evidence of sustained albeit waning neutralization responses from 2003 and 2004. Neutralizing antibodies were detected in samples collected between October 2003 and November 2004. The earlier sample showed binding antibodies to the SARS-CoV-2 S protein and the N proteins of multiple hCoVs. However, the later samples contained antibodies that bound to both the SARS-CoV-2 S and RBD, while exhibiting reduced binding to the N proteins of multiple hCoVs. This transition from antibodies targeting the S protein to those targeting both the S and RBD over time may reflect affinity maturation, a process in which antibodies evolve to bind more specifically and with higher affinity to certain epitopes, such as the RBD without severely impacting neutralization (34, 35). These findings suggest that the individual may have been exposed to a novel coronavirus variant more closely related to SARS-CoV-2, although this does not rule out the possibility of cross-reactivity with other pathogens. For example, previous studies have shown that individuals with malaria or broadly neutralizing antibodies to HIV-1 can cross-reactive to SARS-CoV-2 antigens (36–38). This highlights the complexity of interpreting cross-reactive antibody responses and the need for further investigation into potential sources of exposure and the mechanisms underlying these responses.

It is unknown whether these cross-reactive cellular responses from prior hCoV infection were sustained at levels that would impact the pathogenesis of SARS-CoV-2 during the 2020 pandemic. Immunologic memory to antigenically related infections can result in both positive and negative patient outcomes. As described in influenza, the induction of antibody responses to new virus infection can “backboost” responses to preceding heterologous viruses (39). In contrast, prior heterologous responses with reduced functionality may misdirect new responses resulting in negative clinical outcomes, i.e., “original antigenic sin” (40), particularly in antigenically shifting viruses such as SARS COV-2. Our results show significant prior hCoV infection with demonstrable antibodies to SARS-CoV-2 N and cellular responses to S and N, which when taken in consideration with low rates of severe COVID-19 disease and mortality in Africa, suggest that further research should investigate the possibility that prior hCoV infection provides cross-reactive protective immunity in SARS-CoV-2 infection.

In our risk factor analysis, we saw potential higher odds of SARS-CoV-2 N reactivity, suggesting previous hCoV infection, in women who were > 44 years of age and who had ≥three children. This might be explained by older women having had more children, and therefore increased exposure to respiratory illnesses from young and school-aged children amongst whom susceptibility to infection and transmission may be highest. This hypothesis is supported by studies that have shown that older age is a risk factor for COVID-19, including more severe infections (41, 42). Additionally, a study of household transmission demonstrated while younger children are less likely to be the index COVID-19 patient in the home, they are more likely to be infectious and therefore spread the virus to household members (43). Further studies are warranted to understand the interconnectedness between age and number of children in increased infections by SARS-CoV-2.

Our study of pre-pandemic immunologic reactivity to SARS-CoV-2 in Senegal is unique in its assessment of both antibody and cellular responses. However, our study has limitations. We were unable to evaluate the cellular immune responses to hCoVs to verify the proposed source of SARS-CoV-2 cross-reactive IFN-*γ* cellular responses. Our assessment of primary hCoV infection was based on serologic reactivity to hCoV S, which may not be diagnostic of hCoV infection or correlate with hCoV N reactivity or cross-reactivity to SARS-CoV-2 N. We were unable to directly test our hypothesis by demonstrating that a significant fraction of the population possessed antibody and cellular cross-reactive immune responses to SARS-CoV-2 at the time of the pandemic or by comparing with non-African samples. Also, our study analyzed samples from only adult females, who may have different immune responses than males and children, and from the Dakar region, which may have different circulating hCoVs than other parts of Africa. Finally, our study does not examine immune responses against SARS-CoV-2 variants of concern (VoC). Whether pre-pandemic samples also contain cross-reactive antibody and T cell responses to VoC remains to be determined.

Altogether, our study uniquely analyzed both antibody and cellular responses in Senegalese women, demonstrating that pre-existing immunity against hCoVs can induce cross-reactive T cell responses against SARS-CoV-2. The concurrent measurement of cross-reactive immune responses from both the antibody and cellular arms of the adaptive response provides additional support to the hypothesis that pre-existing hCoV adaptive immunity might impact SARS CoV-2 pathogenesis. These data suggest the need for further studies on cross-reactive SARS-CoV-2 antibody and cellular immunity from hCoVs with more representative geographical and temporal samples as well as comparison with non-African samples. Such studies could provide valuable insights toward a clearer understanding of the dynamics of SARS-CoV-2 disease progression and protection in the West African setting.

## Data Availability

The raw data supporting the conclusions of this article will be made available by the authors, without undue reservation.
