# Supplementary material for: Pre-pandemic cross-reactive antibody and cellular responses against SARS-CoV-2 among female sex workers in Dakar, Senegal
Source: Front Public Health. 2025 Jan 23;13:1522733. doi: 10.3389/fpubh.2025.1522733 (PMC11798920; doi:10.3389/fpubh.2025.1522733)
Supplement: Supplementary file 1 [file Data_Sheet_1.pdf]

Supplementary appendix

Table of Contents **page**

Table S1: Overlap in reactivity between hCoV-229E and hCoV-OC43 …………………………………………….... 2

Table S2: Overlap in reactivity between hCoV-229E and hCoV-HKU1 S ………………………………………….. 2

Table S3: Overlap in reactivity between hCoV-OC43 and hCoV-HKU1 S …………………………………………. 2

Table S1: Overlap in reactivity between hCoV-229E and hCoV-OC43.

		229E				
		**S-only**	**S+N**	**N-only**	**Negative**	**TOTAL**
**OC43**	**S-only**	··	1	··	··	1
	**S+N**	··	5	1	··	6
	**N-only**	2	18	2	3	25
	**Negative**	5	5	··	3	13
	**TOTAL**	7	29	3	6	45

S=spike. N=nucleocapsid.

Table S2: Overlap in reactivity between hCoV-229E and hCoV-HKU1 S.

		229E				
		**S-only**	**S+N**	**N-only**	**Negative**	**TOTAL**
**HKU-1 S**	**Spike-reactive**	7	29	3	6	45
	**Negative**	··	1	··	1	2
	**TOTAL**	7	30	3	7	47

S=spike. N=nucleocapsid.

Table S3: Overlap in reactivity between hCoV-OC43 and hCoV-HKU1 S.

		OC43				
		**S-only**	**S+N**	**N-only**	**Negative**	**TOTAL**
**HKU-1 S**	**Spike-reactive**	1	6	25	13	45
	**Negative**	··	··	1	1	2
	**TOTAL**	1	6	26	14	47

S=spike. N=nucleocapsid.
